# Around the Hospitals

**Published:** 1894-06-09

**Authors:** 


### Around the Hospitals.

[Notice to the Managers of Institutions.—Special space is now reserved for the insertion of notices of hospital meetings and festivals. The Editor requests that all notices may reach The Hospital Office, 428, Strand, at least one week before the date of each meeting.]

**The Oldham Infirmary.**—This institution has been making steady progress during the past year in most respects. All hospitals lament bad times, but in spite of these resources at Oldham are showing an improvement. The adverse balance at the bank has been lessened, annual subscriptions have increased, and substantial legacies have been received. In-patients numbered 645, and out-patients 4,587. The convaescent homes received 46 patients. The income amounted to £4,340, and the expenditure to £5,325. During the year many useful presents were received from the numerous friends of the infirmary.

**The Home Hospitals Association.**—This association, the pioneer in the movement to provide suitable accommodation for sufferers of the wealthier classes, continues to do useful work which is much appreciated, and many more applications for admission are made than can be accepted. The comfort of patients is so well attended to that the balance in hand is always a small one. The inmates mostly enter through the recommendation of medical men, no less than 75 of whom sent patients during the past year. The total number of patients received amounted to 283, and the friends of patients numbered 55. The admission of friends in company with the patients is a great additional privilege, and alone renders the institution of the Fitzroy Home Hospital a great boon. The management is excellent, and the lines upon which the home is conducted are alone a guarantee that patients will receive all the care and comfort possible in return for the moderate payments asked.

**The Queen's Hospital, Birmingham.**—A continuous and increasing strain is thrown on the resources of the Queen's Hospital, an increase in the out-patients' department being especially visible. Twenty-one beds are now reserved for accident cases, a plan that is found to work excellently, as there is now less likelihood that cases urgently needing skilled attention should be turned away. During the year the Misses Stokes, who, with other members of their family, have done so much for Birmingham charities, presented the hospital with a munificient gift of £1,250, to be devoted to permanent improvements. The money has been devoted to the reflooring of the wards with oak, to the introduction of an hydraulic lift, and improving the furnishing of the Nurses' Home. Two hundred pounds yet required to effect these additions was supplied by an anonymous donor. The wards have further been supplied with new bedsteads throughout by four bedstead manufacturing firms and some members of the Committee. The income for 1893 amounted to £7,711, the expenditure to £9,183. After deducting 2s. 6d. per head for each outpatient, numbering 31,736 during the year, the cost per inpatient was £2 14s. 6d.

